# Difference between Cousins

**Published:** 1881-03

**Authors:** 


### DIFFERENCE BETWEEN "COUSINS."

The difference between city and their country cousins is more marked than most people believe. The first impression a man has on finding himself for the first time in a great city is of vague excitement, accompanied by a sense of danger. The multiplicity of objects appear fantastic to an eye accustomed to rural scenery; the unintermittent noises, the entangled yet purposeful movements, and, above all, the shifting panorama of unfamiliar human faces, combine to throw the visitor into a state of mind totally strange to him.

And amid so much tumultuous life he sees death everywhere on the lookout for a victim. But if the visitor to these strange regions looks at the faces of those he meets in search of some reflection of his own perturbation, he looks in vain.

The countenance of the city man, as he threads his way along the streets, is curiously impassive. At a first glance it appears also to be unobservant; but this is not it. For though he seems to look at nothing, it soon becomes evident that he sees everything.

He mechanically informs himself out of the corner of his eye, of everything that might tend to obstruct or threaten him; and though he passes through a thousand people without encountering the gaze or treading on the toes of any one of them, he will recognize an acquaintance or calculate to an inch the rate of speed at which he must make the crossing in order to escape the omnibus from one direction and the truck from the other.

Doubtless custom and memory will account for a large part of it; yet the impassive face would probably appear far less impassive than it does had not the contraction of the facial muscles, brought about about by the constant assaults of innumerable impressions and the impossibility of responding to them all, become in a manner fixed.

The houses and the pavements, the vehicles and the hub-bub, produce an effect upon these muscles just the reverse of that exercised by the hills and dales of the country; they press them in instead of drawing them out—in other words, the mind resists them instead of sympathizing with them.

---

IT is claimed that a man never loses anything by politeness, but this has proved to be a mistake. An old Philadelphian lifted his hat to a young lady, and the wind carried away his wig.

